# Fetal Hydantoin Syndrome and Its Anaesthetic Implications: A Case Report

**DOI:** 10.1155/2012/370412

**Published:** 2012-10-02

**Authors:** Ranju Singh, Nishant Kumar, Sakshi Arora, Ritu Bhandari, Aruna Jain

**Affiliations:** Department of Anesthesiology and Critical Care, Lady Hardinge Medical College and Associated Hospitals, New Delhi 110001, India

## Abstract

Fetal hydantoin syndrome is a rare disorder that is believed to be caused by exposure of a fetus to the anticonvulsant drug phenytoin. The classic features of fetal hydantoin syndrome include craniofacial anomalies, prenatal and postnatal growth deficiencies, underdeveloped nails of the fingers and toes, and mental retardation. Less frequently observed anomalies include cleft lip and palate, microcephaly, ocular defects, cardiovascular anomalies, hypospadias, umbilical and inguinal hernias, and significant developmental delays. Anaesthesia for incidental surgery in such a patient poses unique challenges for the anesthesiologist. We report the successful management of a 4-year-old male child with fetal hydantoin syndrome, cleft palate, spina bifida, atrial septal defect, and dextrocardia for tibialis anterior lengthening under subarachnoid block.

## 1. Introduction

Patients with multiple congenital anomalies often have special needs requiring attention and present unique challenges to the health care provider responsible for administering sedation and anesthesia. It is important to recognize risk factors and potential complications before anesthetizing these patients. The association among maternal epilepsy, anticonvulsant drugs, and an increased incidence of congenital abnormalities has been suspected for at least 25 years. Although, most anticonvulsant medications have been implicated as potential teratogens, phenytoin, valproic acid, carbamazepine, or a combination therapy with these compounds is involved most commonly [1, 2]. We report the successful management of a 4-year-old male child with fetal hydantoin syndrome, spina bifida, and dextrocardia for bilateral tibialis anterior lengthening under subarachnoid block.

## 2. Case Report

A four-year-old, 20 kg male child with congenital talipes equinovarus was posted for bilateral tibialis anterior lengthening surgery. The patient's mother gave a history of being an epileptic and was taking tablet phenytoin 100 mg BD for the last 7 years and throughout the antenatal period. The mother was attending an antenatal clinic, where she was advised to continue with the drug therapy in view of persistent convulsions. She had had a previous caesarean section and the child was born healthy and devoid of any defects. This child was born at term through a caesarean delivery in view of nonprogress of labor. The child developed seizures on first day of life and had further episodes in February and June 2011, but was not advised any medication. The child had history of recurrent upper respiratory tract infections and delayed milestones. He had abnormal facies, webbed fingers, low set ears, and hypertelorism. He also had an incomplete cleft palate and spina bifida occulta of L5. The chest was bilaterally clear with no murmurs on auscultation. There was no neurological deficit. Routine blood investigations were within normal limits. The X-ray chest revealed dextrocardia. The child was then further investigated and an EEG, ECG, MRI skull and lumbar spine, and echocardiogram were carried out. EEG revealed abnormal spikes in the frontoparietal region. The MRI showed cystic encephalomalacia in bilateral peritrigonal and periventricular deep white mater which was suggestive of prenatal hypoxic ischemic encephalopathy, whereas examination of the spine showed a bony deformity of the L5 spine without involvement of the spinal cord or nerves. ECG showed normal sinus rhythm and echocardiogram revealed ostium secundum atrial-septal defect, 4.8 mm in size with no left-to-right shunt and an ejection fraction of 62%.

On the day of surgery, the patient was premedicated with oral midazolam 0.5 mg kg^−1^ (10 mg) 30 minutes prior to surgery and EMLA cream was applied on the dorsum for canulation. On the operation table, ECG, NIBP, and SpO_2_ were attached. A 22 G intravenous line was secured and infusion of propofol at 75 *μ*g/kg/min was started for sedation. The patient was then turned to left lateral position and under all aseptic precautions a subarachnoid block was given at L3-L4 with 1.2 mL of 0.5% hyperbaric bupivacaine and 10 *μ*g of fentanyl with a 25 G 5 cm Quincke disposable paediatric spinal needle. The patient was carefully turned to the supine position and oxygen supplementation started with a face mask. The patient remained haemodynamically stable throughout procedure which lasted for 1.5 hrs. Postoperatively, the child was shifted to the postanaesthesia care unit for a period of 1 hour and thereafter shifted to ward. There was no postoperative sensorimotor deficit. 

## 3. Discussion

Fetal hydantoin syndrome is a fetopathy likely to occur when a pregnant patient takes phenytoin for epileptic seizures. Phenytoin is a known teratogen and the FDA has labeled phenytoin as a category D medication because of its potential to cause significant, unreasonable harm to a fetus. Approximately, 2% of women giving birth are epileptic and phenytoin is prescribed in 5%−20% of patients. The risk that an infant exposed to hydantoin in utero will have the clinical phenotype of the full-blown fetal hydantoin syndrome is approximately 5%–10%, whereas the risk of an infant's expressing some effects of the syndrome is 33% [3, 4]. Phenytoin interferes with the body's ability to absorb folic acid. Without an adequate supply of folic acid, infants have a significantly increased risk of developing major birth injuries.

In utero, exposure to this drug causes characteristic dysmorphic syndrome in the newborn like low set and malformed ears, short neck, deep nasal bridge, hypertelorism, and hypoplastic distal phalanges of fingers and toes. These dysmorphic features are often associated with growth retardation and delayed psychomotor development [3, 4]. The risk of neurological impairment estimated to be 1% to 11% is 2 to 3 times higher than in the general population. The risk of oral clefts and cardiac anomalies is 5 times than others in hydantoin exposed infants. Less frequently observed abnormalities include microcephaly, ocular defects, hypospadias, umbilical and inguinal hernias [3, 4]. Increased incidence of benign and malignant neurological tumors has also been suggested.

A baby with fetal hydantoin syndrome can present to an anesthesiologist for an orthopedic surgery, neurosurgery, cleft lip or cleft palate surgery, or for any emergency surgery. Items that may have an impact on the delivery of care to the patient include a difficult airway as a result of upper or lower airway obstruction, short neck, or defects (e.g., cleft lip/palate, small chin or mouth, craniofacial deformities); altered respiratory mechanisms caused by skeletal anomalies or cardiovascular disorder (arrhythmia or structural defect); neuromuscular problems (e.g., myotonia, muscular dystrophy or weakness; central nervous system defects). Special attention should be given to liver and renal problems when sedation/anesthetics are used. 

We decided to proceed with regional anaesthesia in this case in view of reactive and a potentially difficult airway, requirement of the surgical procedure and effective postoperative analgesia. Although general anaesthesia was an alternative, possible reversal of shunt, prolonged postoperative fasting, pain, nausea, vomiting, and inability of the child to communicate effectively were factors which tilted the balance in favor of regional anaesthesia. We opted for a subarachnoid block instead of caudal epidural in view of spina bifida. Since the child was mentally retarded and it was difficult to coerce his cooperation, sedation with propofol infusion was instituted. 

Congenital disorders pose a significant challenge to the anaesthesiologists. Central neuraxial blockade, though relatively contraindicated in the presence of spina bifida, should not be withheld in the absence of involvement of the spinal cord or sensorimotor deficit. Understanding the unique anesthesia considerations can help avoid morbidity and mortality in such patients. 
